# Paeonol suppresses solar ultraviolet-induced skin inflammation by targeting T-LAK cell-originated protein kinase

**DOI:** 10.18632/oncotarget.15636

**Published:** 2017-02-23

**Authors:** Peipei Xue, Yong Wang, Fanfan Zeng, Ruijuan Xiu, Jingwen Chen, Jinguang Guo, Ping Yuan, Lin Liu, Juanjuan Xiao, Hui Lu, Dan Wu, Huaxiong Pan, Mingmin Lu, Feng Zhu, Fei Shi, Qiuhong Duan

**Affiliations:** ^1^ Department of Biochemistry and Molecular Biology, School of Basic Medicine, Huazhong University of Science and Technology, Wuhan, 430030, China; ^2^ Department of Dermatology of The General Hospital of Air Force, Beijing, 100142, PR China; ^3^ Department of Pathology, Union Hospital, Huazhong University of Science and Technology, Wuhan, 430030, China

**Keywords:** TOPK, paeonol, solar UV, skin inflammation, MAPK

## Abstract

Excessive exposure to solar UV (SUV) is related with numerous human skin disorders, such as skin inflammation, photoaging and carcinogenesis. T-LAK cell- originated protein kinase (TOPK), an upstream of p38 mitogen-activated protein kinase (p38) and c-Jun N-terminal kinases (JNKs), plays an important role in SUV -induced skin inflammation, and targeting TOPK has already been a strategy to prevent skin inflammation. In this study, we found that the expression of TOPK, phosphorylation of p38 or JNKs was increased in human solar dermatitis tissues. The level of phosphorylation of p38 or JNKs increased in a dose and time dependent manner in HaCat cells or JB6 Cl41 cells after SUV treatment. Paeonol is an active component isolated from traditional Chinese herbal medicines, and MTS (3-(4,5-dimethylthiazol-2-yl)-5-(3-carboxymethoxyphenyl)-2H-tetrazdium) assay showed that it has no toxicity to cells. Microscale thermophoresis (MST) assay showed that paeonol can bind TOPK *ex vivo*. *In vitro* kinase assay showed paeonol can inhibit TOPK activity. *Ex vivo* studies further showed paeonol suppressed SUV-induced phosphorylation level of p38, JNKs, MSK1 and histone H2AX by inhibiting TOPK activity in a time and dose dependent manner. Paeonol inhibited the secretion of IL-6 and TNF-α in HaCat and JB6 cells *ex vivo*. *In vivo* studies demonstrated that paeonol inhibited SUV-induced increase of TOPK, the phosphorylation of p38, JNKs and H2AX, and the secretion of IL-6 and TNF-α in Babl/c mouse. In summary, our data indicated a protective role of paeonol against SUV-induced inflammation by targeting TOPK, and paeonol could be a promising agent for the treatment of SUV-induced skin inflammation.

## INTRODUCTION

Exposure to ultraviolet (UV) irradiation causes DNA damage and other cellular responses that contribute to numerous human skin disorders, such as skin inflammation, photoaging, and carcinogenesis [1–3]. Depending on wavelength, UV radiation in sunlight can be divided into short-wave UVC (200-280 nm), mid-wave UVB (280-320 nm) and long-wave UVA (320-400 nm) [4]. All UVC and most of UVB (95%) can be absorbed by the ozone layer efficiently [5]. Both UVA and UVB are harmful to human skin. Despite major advances in our understanding of skin biology, the incidence of skin disorder, especially of skin inflammation and skin cancer, is reaching epidemic proportions, indicating that more effective strategies and innovative new treatments are required.

T-LAK cell-originated protein kinase (TOPK) was first studied as a novel MAPKK-like protein kinase [6] in 2000. It is highly expressed in a variety of tumors including breast cancer [7], colorectal cancer [8], lung cancer [9] and hepatocellular carcinoma [10]. Activation of TOPK is closely related to tumor development. In recent years, it has been shown that TOPK is closely related to Solar UV (SUV) -induced skin inflammation and considered to be an effective therapeutic target for SUV-induced skin inflammation [11, 12].

Paeonol is isolated from traditional Chinese herbal medicines, such as moutan cortex [13], roots of Paeonia lactiflora Pallas [14] and Dioscorea japonica, and has been used for thousands of years in China. Paeonol has a wide range of biological effects, including anti-inflammatory, immune regulatory, anti-tumor, and anti-oxidative effects [15, 16]. Paeonol is used in food additive and traditional oriental medicines in treating various diseases including Alzheimer's diseases [17, 18] and atherosclerosis [19]. Recently it is reported that paeonol suppresses lipopoly- saccharide-induced inflammatory cytokines in macrophage cells [20] and attenuates airway inflammation and hyper-responsiveness in a murine model [21]. Therefore, paeonol may be a potential therapeutic agent for treating SUV-induced skin inflammation.

In this study, we found that paeonol could suppress SUV-induced skin inflammation by targeting TOPK, and therefore may be a potential preventive agent for SUV-induced skin inflammation.

## RESULTS

### The phosphorylation level of p38 and JNKs were increased in a dose and time dependent manner in JB6 Cl41 and HaCat cells after SUV irradiation

Previous reports have showed that TOPK is closely associated with SUV induced skin inflammation [22]. JNKs and p38 are the two major subgroup kinases of the MAPK family, which can be activated by SUV irradiation [23]. First six cases of solar dermatitis and normal skin samples were detected to observe pathological changes in solar dermatitis. The data (See Supplementary Figure 1) indicated that SUV could induce elevated TOPK and mitogen-activated protein kinases (MAPKs), such as phosphorylated p38 and JNKs accompanied with skin inflammation.

**Figure 1:**
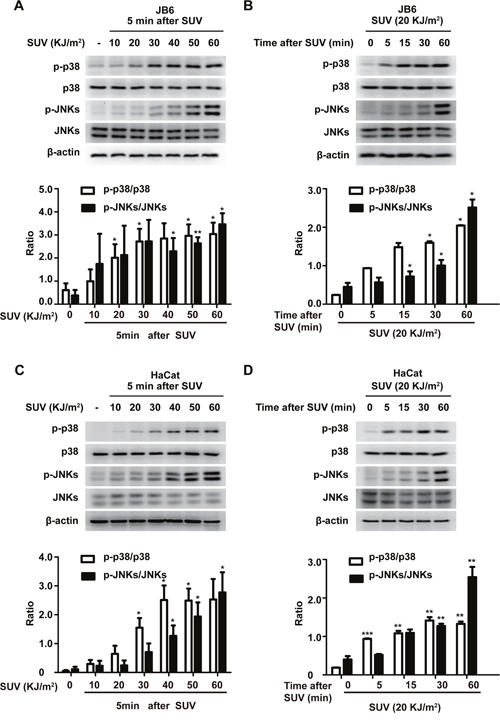
SUV irradiation induces the phosphorylation of p38 and JNKs in a dose- and time-dependent manner in JB6 Cl41 and HaCat cells **A**. Phosphorylation of p38 or JNKs in JB6 Cl41 cells was greatly activated by SUV as indicated in a dose-dependent manner. **B**. Phosphorylation of p38 or JNKs in JB6 Cl41 cells was greatly activated by SUV as indicated in a time-dependent manner. **C and D**. HaCat cells were treated in the same manner as JB6 Cl41 cells. The cell lysates were subjected to 10% SDS-PAGE. Protein bands were detected by Western blot. Pictures shown are representative results from at least triplicate experiments. Histograms shown are the statistical results of at least three independent experiments. The asterisks indicated a significant difference compared with 0 group (*P<0.05, **P<0.01, ***P<0.001).

Mouse epidermal JB6 Cl41 cell line and Human keratinocyte HaCat cell line were often used as cellular models to study SUV-induced skin inflammation [23, 24]. SUV-activated signaling transduction pathways are mediated primarily through signaling cascades involving MAPKs, including JNKs and the p38 kinases [25, 26]. The level of phosphorylation of JNKs and the p38 kinases can reflect the efficiency of established UV System in our lab. First, the levels of phosphorylated p38 and JNKs were examined after UV irradiation in JB6 cells. With the dose of UV increased from 10 to 50 KJ/m^2^, the level of phosphorylated p38 and JNKs gradually increased (Figure 1A). The level of phosphorylated p38 and JNKs reached its peak at 5 min after 20 KJ/m^2^ SUV irradiation. Next, the level of phosphorylated p38 and JNKs gradually increased as well from 5min to 60 min after 20 KJ/m^2^ SUV irradiation (Figure 1B). Similar results were found in HaCat cells (Figure 1C, 1D). These data indicated that the UV system was reliable, and the following experiments were performed using the UV system.

### Paeonol binds with TOPK and inhibits TOPK activity

**Figure 2:**
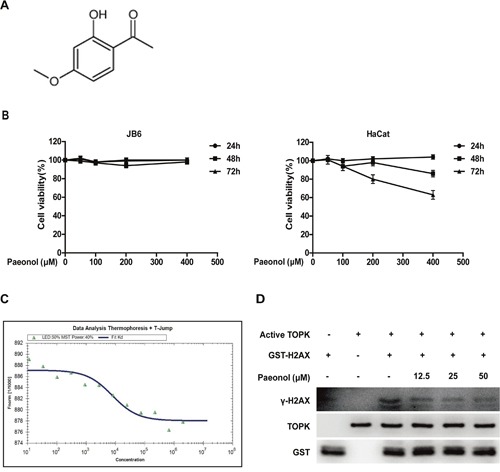
Paeonol binds with TOPK and inhibits TOPK activity **A**. The chemical structure of paeonol. **B**. HaCat cells and JB6 cells were treated with 50, 100, 200, and 400 μM of paeonol for 24 h, 48h, and 72h. The cell viability was determined by MTS assay according to the manufacturer's instructions. Data are shown as means ± standard deviation from at least three independent experiments. **C**. The affinity between paeonol and TOPK was measured with MST assay. The resulting binding curve was shown with a Kd value of 7670+/−690 nM. **D**. The activity of TOPK was inhibited by paeonol in a dose-dependent manner *in vitro*.

On the basis of comprehensive spectral analysis and data published previously, Paeonol was elucidated as a known 1-(2-hydroxy-4-methoxyphenyl) ethanone (Figure 2A). To determine the cytotoxicity of paeonol, HaCat and JB6 Cl41 cells were treated with different concentrations (0, 50, 100, 200 and 400 μM) of paeonol for different time (24, 48 and 72 h) and measured using MTS (3-(4, 5-dimethylthiazol-2-yl)-5-(3-carboxymethoxyphenyl)-2H-tetrazdium) assay. The results indicated that paeonol had no significant cytotoxicity on JB6 Cl41 and HaCat cells (Figure 2B). TOPK has already been regarded as a therapeutic target for SUV-induced skin inflammation and paeonol has anti-inflammatory activities. Therefore, it would be very interesting to test if paeonol can bind with TOPK. Microscale thermophoresis (MST) assay is an optical method which works in free-solution and with low consumption of sample. It can quantify protein-protein interactions or protein-small molecule interactions with high sensitivity [27]. MST assay was performed to test the affinity between paeonol and TOPK. In the study, paeonol exhibited a quite low equilibrium dissociation constant (Kd) of 7670+/−690 nM, which indicated a strong affinity with TOPK (Figure 2C). *In vitro* kinase assay showed that when the concentration of paeonol increased from 12.5 μM to 50 μM, the level of γ-H2AX catalyzed by active TOPK gradually decreased (Figure 2D). These data indicated that paeonol could bind with TOPK and inhibit its activity, and have no significant cytotoxicity.

### Paeonol down-regulates SUV-induced activation of p38 and JNKs

**Figure 3:**
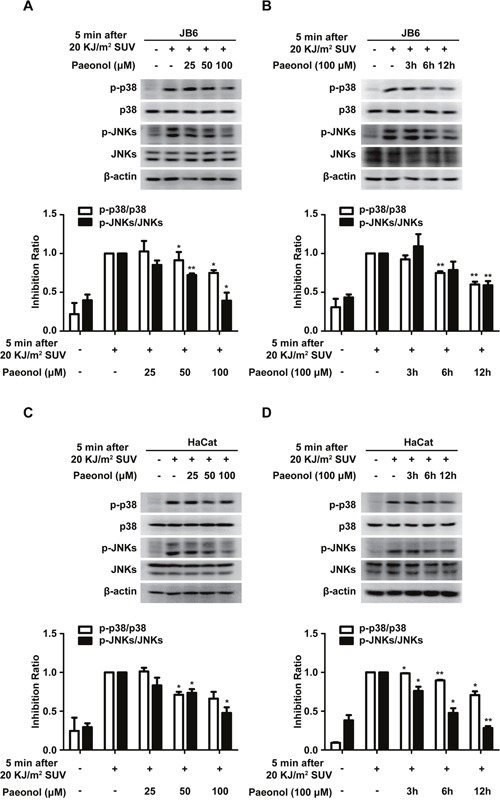
Paeonol down-regulates SUV-induced activation of p38 and JNKs **A** and **B**. Phosphorylation of p38 or JNKs irradiated by SUV was substantially attenuated in a dose- and time-dependent manner after pretreated with paeonol in JB6 Cl41 cells. **C** and **D**. HaCat cells were treated in the same conditions as in JB6 Cl41 cells. Data shown are representative results from at least triplicate experiments. The asterisks indicated a significant difference compared with the group treated with 20KJ/m^2^ SUV (*P<0.05, **P<0.01).

Next, paeonol was tested whether it can block TOPK activity *ex vivo*. JB6 cells were pretreated with paeonol for 12 hours from 25 μM to 100 μM before 20 KJ/m^2^ SUV irradiation, and the level of phosphorylated p38 or JNKs was tested. The results demonstrated that the level of phosphorylated p38 or JNKs gradually decreased (Figure 3A). Next, the level of phosphorylated p38 or JNKs also gradually decreased in JB6 cells with the incubation time increased from 3 hours to 12 hours incubated with 100 μM paeonol before 20KJ/m^2^ SUV irradiation (Figure 3B). Similar results were obtained in HaCat cells (Figure 3C, 3D). These data indicated that paeonol could suppress activation of p38 or JNKs induced by SUV *ex vivo*.

### Paeonol down-regulates SUV-induced downstream TOPK signaling pathway in a dose- and time-dependent manner and inhibits the secretion of cytokines in the JB6 Cl41 and HaCat cells

**Figure 4:**
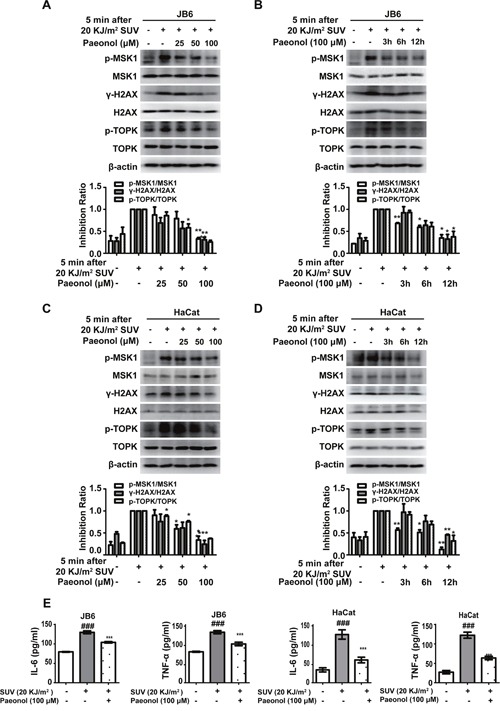
Paeonol down-regulates SUV-induced downstream TOPK signaling pathway in a dose- and time-dependent manner and inhibits the secretion of cytokines in the JB6 Cl41 and HaCat cells **A** and **B**. Phosphorylation of MSK1, H2AX, or TOPK was substantially attenuated in a dose- and time-dependent manner after pretreated with paeonol irradiated by SUV in JB6 Cl41 cells. Cells were pre-treated with paeonol and then stimulated with SUV as indicated. **C** and **D**. HaCat cells were treated in the same manner as in JB6 cells. Data shown are representative results from at least triplicate experiments. **E**. Paeonol inhibited the secretion of IL-6 and TNF-α induced by SUV. The asterisks indicated a significant difference compared with the group treated with 20KJ/m^2^ SUV (* *P<0.05*, ** *P<0.01*, *** *P<0.001*). The marks indicated a significant difference compared with control group(*^###^P<0.001*).

Histone H2AX is one of the substrates of TOPK and can be phosphorylated at Ser-139 (γ-H2AX) by TOPK [28]. γ-H2AX is a biomarker for DNA double-strand breaks [29]. TOPK is also an upstream kinase of p38. MSK1 is a downstream substrate of p38 kinase and also related to DNA damage. In the present study, the level of phosphorylated MSK1, γ-H2AX or TOPK gradually decreased after pretreated with paeonol from 25 μM to 100 μM for 12 hours in JB6 cells before 20 KJ/m^2^ SUV irradiation (Figure 4A). Next, the level of phosphorylated MSK1, H2AX or TOPK gradually decreased after pretreated with 100 μM paeonol from 3 hours to 12 hours before 20 KJ/m^2^ SUV irradiation in JB6 cells (Figure 4B). Similar results were observed in HaCat cells (Figure 4C, 4D). JNKs and p38 kinases are involved in modulation of inflammatory responses through up-regulating the expression of cytokines. Cytokines, such as TNF-α and IL-6, played important roles in inflammation [30]. In this study, the increase of secretion of TNF-α or IL-6 irritated by 20 KJ/m^2^ SUV was inhibited by 100 μM paeonol in JB6 or HaCat cells (Figure 4E). These results indicated that paeonol could suppress SUV-induced DNA damage through inhibiting TOPK activity, and the secretion of cytokines was inhibited by paeonol *ex vivo*. Therefore, the inflammation induced by SUV could be inhibited by paeonol *ex vivo*.

### SUV-induced inflammation was inhibited by paeonol *in vivo*

**Figure 5:**
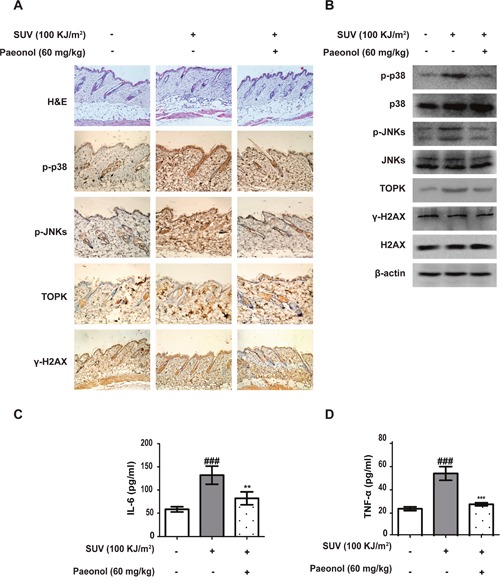
SUV-induced inflammation was inhibited by paeonol *in vivo* **A**. SUV-induced inflammation, phosphorylation of p38 and JNKs, TOPK level, and γ-H2AX was inhibited by paeonol treatment in mouse skin. The sections of mouse skin were stained with H&E and IHC. **B**. The level of TOPK, p38, p-p38, JNKs, p-JNKs, H2AX and γ-H2AX in mouse skin tissues after SUV irradiation were detected by western blot. **C** and **D**. The secretion of IL-6 or TNF-α was obviously reduced by paeonol treatment in mouse skin. Data shown were expressed as mean ± SEM. The marks indicated a significant difference compared with control group (^###^*P<0.001*; The asterisks indicated a significant difference compared with SUV-irradiated group (**P<0.01, ***P<0.001).

Next, whether paeonol can inhibit skin inflammation induced by SUV *in vivo* was tested. First, after 100 KJ/m^2^ SUV irradiation, epidermal hyperkeratosis, infiltration of inflammatory cells, and multifocal intercellular edema were observed in mouse skin tissue using H&E staining. They were all signs of skin inflammation. Second, compared with control group, the level of TOPK, phosphorylated p38, phosphorylated JNKs and γ-H2AX in mouse skin tissue was increased after irradiation (Figure 5A and 5B). Third, the concentration of IL-6 and TNF-α secreted by mouse skin tissue were increased after irradiation, and paeonol (60mg/kg) could inhibit it after smeared on the mouse skin before irradiation (Figure 5C). These data indicated paeonol could inhibit SUV-induced skin inflammation and DNA damage *in vivo*.

## DISCUSSION

Excessive exposure to UV can induce skin inflammation. TOPK could be a therapeutic target for SUV; induced skin inflammation. SUV, especially UVB exposure results in several types of DNA damage, such as the formation of 8-hydroxy-2′-deoxyguanosine (8-OHdG), cyclobutane pyrimidine dimers, and pyrimidine (6-4) pyrimidone photoproducts [30–32]. These DNA damages can in turn lead to skin inflammation, a process that precedes photoaging and carcinogenesis [33]. MAPKs are a family of proteins that can be activated by diverse extracellular stimuli, such as growth factors, UV, and DNA damage. Upon activation of the MAPKs, transcription factors present in the cytoplasm or nucleus are phosphorylated and activated, leading to the expression of target genes resulting in biological responses such as proliferation, apoptosis, and inflammation [23]. The JNKs and the p38 kinases are two major subgroups of the MAPK family. TOPK is the upstream of the JNKs and the p38 kinases [34, 35] and contributes to the generation of γ-H2AX [36], a biomarker for DNA double-strand breaks. MAPK inhibitors as anti-inflammatory drugs can reduce both the synthesis of pro-inflammatory cytokines and their intracellular signaling, but they possess poor specificity and potential side effects (mainly hepatotoxicity) during chronic treatment [37].

Since TOPK is the upstream of the JNKs and the p38 kinases and contributes to the formation of γ-H2AX, TOPK inhibitors not only can inhibit the activation of MAPKs, but also reduce the inflammation caused by DNA damage. Moreover, TOPK inhibitors may have fewer side effects because the ablation of TOPK does not affect normal cell cycle progression [36] and the level of TOPK is very low in normal skins (Supplementary Figure 1B). Therefore, TOPK inhibitors are very promising to prevent skin inflammation caused by solar UV.

Traditional Chinese Medicine (TCM) is a kind of traditional Asian medicine that has evolved over more than 2500 years. It includes various forms of herbal medicine, acupuncture, massage (Tuina), exercise (Qigong) and dietary therapy. It has helped the ancient Chinese to deal with many kinds of diseases. In TCM, typical prescription also called formulae, consist of several kinds of medicinal herbs or minerals, in which one represents the principal component, the others strengthen the roles of the principal one. In some formulae, multiple components could exert synergistic therapeutic efficacies by hitting multiple targets. And sometimes, a single compound might even treat a specific disease. For example, artemisinin is currently the most effective treatment for malaria. However, in most formulae, essential compounds and its targets remain to be addressed by modern molecular approaches. Nevertheless, TCM has become an essential component of the current medical system [38] and can be used as a treasury to explore novel compounds. Paeonol, a compound isolated from traditional Chinese herbal medicines which are all plants in nature and used for thousands of years safely, possessed anti-inflammatory activity [39, 40]. Up to now, there have been no reports associated with the side effect of paeonol, although it is possible that a few people may be allergic to the topical use of paeonol. Moreover, it has been reported that paeonol could reduce the production of TNF-α and IL-6 via inactivation of IκBα, ERK1/2, JNK, and p38 MAPK [41]. Both TNF-α and IL-6 are inflammatory mediators which contribute to the development of inflammation [42]. In the present study, we found paeonol as a novel TOPK inhibitor from TCM, could suppress SUV-induced skin inflammation.

Topical steroids are a form of corticosteroids as a cream or ointment for the treatment of rash, eczema, and dermatitis. Previous studies showed that glucocorticosteroids had great anti-inflammatory and immunosuppressive activities [43, 44], which provided insight into their model of efficacy. Glucocorticosteroids first integrated with cytosolic glucocorticoid receptors, which then transferred to the nucleus and regulated gene expression of targeted cells [45]. Topical glucocorticoid therapy is one of the most commonly used anti-inflammatory drugs with invaluable therapeutic efficacies, but it has many adverse effects such as atrophy, striae, rosacea, perioral dermatitis, acne, and purpura [46]. It is urgent to find non-steroid drugs with fewer side effects that can inhibit skin inflammation. In this study, we found paeonol with little side effects, a compound purified from traditional Chinese herbal medicine, could suppress SUV-induced skin inflammation.

Besides TOPK, monoamine oxidase (MAO) is another target of paeonol [47], although there is no report that any other kinase of activity could be inhibited by paeonol. MAO is an important enzyme catalyzing the metabolism of many endogenous monoamine neurotransmitters such as noradrenalin, dopamine, and serotonin (5-HT). Some MAO A inhibitors are used to treat anxiety and depression, while some MAO B inhibitors appear to be effective to treat Parkinson's disease [48]. Moreover, some MAO inhibitors have been found to be clinically effective for skin diseases such as psoriasis and atopic dermatitis by lowering the levels of TNF-α [49, 50]. Both MAO A and MAO B were found to be the targets of paeonol and the activities of them can be inhibited by paeonol [48]. Since paeonol can inhibit skin inflammation by inhibiting TOPK, it would be very interesting to investigate the role of MAO family further in solar UV induced skin inflammation.

In conclusion, our results indicated that paeonol can suppress SUV-induced skin inflammation by targeting TOPK *in vitro* and *in vivo*. It can be a promising ingredient to prevent SUV-induced skin inflammation.

## MATERIALS AND METHODS

### SUV device, agents and antibodies

The SUV lambs used in this study were purchased from Q-Lab Corporation (Cleveland, OH). The percentage of UVA and UVB emitted from SUV lamps was measured by a UV radiometer and was recorded as 92.5% and 7.5%, respectively. Paeonol (the purity >99%) was purchased from Chengdu Ruifensi Biotechnology Co. Ltd (Chengdu, China). The pGEX-GST-H2AX plasmid was purchased from Addgene Inc. The active TOPK was purchased from Millippore Company (Billerica, MA, USA). Both the TNF-α and IL-6 ELISA kits were purchased from Dakewe Biotech Co. Ltd (Shenzhen, China). The primary antibodies for TOPK, JNKs, p38, H2AX, p-TOPK (Thr9), p-JNKs (Thr183/Tyr185), p-p38 (Thr180/Tyr182), and γ-H2AX (Ser139) were purchased from Cell Signaling Technology (USA). The primary antibody for β-actin was obtained from Santa Cruz (USA). Horseradish peroxidase (HRP)-conjugated Goat anti Mouse IgG (H+L) and Goat anti Rabbit IgG (H+L) secondary antibodies were purchased from Earth Ox life sciences company (San Francisco US).

### Cell culture

The human skin keratinocyte HaCat cell line and the mouse epidermal JB6 Cl41 cell line obtained from American Type Culture Collection (ATCC, USA), were cultured and used following the procedures provided by ATCC. HaCat cells were cultured in Dulbecco's modified Eagle's medium (DMEM) containing 10% fetal bovine serum (FBS), while JB6 Cl41 cells were in Eagle's minimum essential medium (MEM) containing 5% fetal bovine serum (FBS). These cells were cultured in a 37°C, 5% CO_2_ incubator.

### MTS assay

MTS assay was employed to test cell viability. Cells were first seeded in 96-well plates (1000/well) overnight, then treated with different concentrations of paeonol (0, 50, 100, 200, 400 μM) for different time (24, 48, or 72 h). Cell viability was measured with an MTS assay kit (Promega, Madison, WI) according to the manufacturer's instructions, and the absorbance was read at 490 nm.

### MST assay

Paeonol stock was dissolved in ddH_2_O in a concentration of 4 mM. We used 4 mM paeonol as the highest concentration for the serial dilution. According to labeling protocol, recombinant TOPK was labeled with the Monolith NT™ Protein Labeling Kit RED. Labeled TOPK was used in a concentration of 200 nM. After 10 minutes incubation at room temperature, the samples were loaded into MonolithTM standard-treated capillaries and the thermophoresis was measured at 25°C after 30 minutes incubation on a Monolith NT.115 instrument (NanoTemper Technologies, München, Germany). The dissociation constant Kd values were fitted by using the NTAnalysis software.

### Western blot

HaCat cells or JB6 Cl41 cells were seeded in 6-cm dishes. After 24 hours’ culture and 12hours’ starvation, they were stimulated with SUV irradiation. The cells were irritated with different doses of SUV (0, 10, 20, 30, 40, 50, 60KJ/m^2^) or harvested after different delay time (0, 5, 15, 30, 60 min). In order to study the effect of paeonol, the cells first were starved in serum-free medium for 12 h, and then pretreated with paeonol (25, 50, 100 μM) for 3, 6, or 12h before SUV irradiation. After harvested and disrupted in lysis buffer, the cells were sonicated for 45 seconds and centrifugated at 12,000 rpm for 10 min. The protein concentrations of the samples were determined with Bradford method. The samples were first diluted with 5× SDS loading buffer in proper proportion, and then heated at 95°C for 10 minutes. The samples (20-60μg) were separated by SDS-PAGE and then transferred to polyvinylidene fluoride (PVDF) membranes. The membranes were blocked with 5% non-fat milk or 5% BSA in TBST. After that, they were incubated with a specific primary antibody at 4°C overnight. The ECL system (BIO-RAD, USA) was used to visualize the protein bands. All results obtained were from at least triplicate independent experiments.

### Prokaryotic expression and purification of GST-H2AX fusion protein

E. ColiBL21 bacteria were used to express the human GST-H2AX fusion protein. When the bacteria grew at 37°C in a constant temperature culture shaker to an absorbance of 0.8-0.9 at 600 nm, they were induced with 1 mM isopropyl-β-D-thiogalactopyranoside (IPTG) for another 1-2 hours at 37°C and then harvested by centrifugation. After repeated freezing and thawing, the cell pellets were suspended in Phosphate Buffered Saline (PBS). The cell lysates were sonicated for 20 minutes with maximum intensity on ice and centrifuged for 10 minutes at a speed of 3000 rpm. Then the supernatant fraction of them was incubated with Glutathione-Sepharose beads (GE, USA) at 4°C overnight. The beads were washed with PBS for three times and eluted with 50 mM Glutathione.

### *In vitro* Kinase assay

GST-H2AX proteins, active TOPK, and ATP were used for the *in vitro* kinase assay. Reactions were conducted in 1×kinase buffer containing 100 μM ATP. After incubated at 30°C for 30 minutes, the reaction was stopped by 5×SDS loading buffer and the mixture was separated by SDS-PAGE. Phosphorylated H2AX, total H2AX and total TOPK were detected respectively.

### Animal study

Thirty male Balb/c mice (6-weeks-old) were purchased from the Center for Disease Control and Prevention in Hubei province (Hubei, China). They were all kept on a 12 h light/dark cycle at a controlled temperature with free access to food and tap water for a week and then shaved 24 h before experiment. The mice were randomly divided into three groups: vehicle group (n=10), SUV group (n=10), paeonol (60mg/kg) group (n=10). The mice were shaved 24 h before experiment. In the vehicle group, the dorsal skin of mice was smeared with acetone for 3 h. In the SUV group, the dorsal skin of mice was smeared with acetone for 3 h and then exposed to 100 KJ/m^2^ SUV. In paeonol (60mg/kg) group, 60 mg/kg paeonol in acetone was smeared to the dorsal skin for 3 h and mice were exposed to 100 KJ/m^2^ SUV. The mice were euthanized and dorsal trunk skin samples were harvested at 24 h after SUV irradiation. One-half of the samples were immediately fixed in 4% paraformaldehyde and for hematoxylin and eosin (H&E) staining and immunohistochemistry (IHC). The other samples were put in a -80°C freezer. Before used, they were placed at room temperature for 30 minutes. After that, they were added 1×PBS proportionally, homogenized and centrifuged. The supernatant were collected and used for ELISA assay and Western blot assay. All animal studies were conducted according to the guidelines approved by the Laboratory Animal Center of Huazhong University of Science and Technology.

### IHC

Antigen retrieval was conducted in both human and mouse skin sections (5μm) with microwave after deparaffinization and rehydration for 10 min in sodium citrate buffer. Then 3% H_2_O_2_ was used to deal with the sections for 10 min. Next, the sections were blocked with 5% goat serum for 1 h at room temperature. And then the sections were incubated with their corresponding primary antibodies at 4°C overnight. A biotinylated-streptavidin-HRP and DAB system was used for color reaction. All sections were counter-stained with hematoxylin. Images from immunostaining were analyzed by Medicine Image Analysis System (MIAS).

### Statistical analysis

All quantitative data are reported as means ± SD. Significant differences were determined by *t* test or one-way ANOVA. *P<0.05* was considered to be significant (* or ^#^ *P* < 0.05, ** or ^##^*P* < 0.01, *** or ^###^ *P* < 0.001).

## SUPPLEMENTARY FIGURE


