# Supplementary material for: Paeonol suppresses solar ultraviolet-induced skin inflammation by targeting T-LAK cell-originated protein kinase
Source: Oncotarget. 2017 Feb 23;8(16):27093–104. doi: 10.18632/oncotarget.15636 (PMC5432320; doi:10.18632/oncotarget.15636)
Supplement: Supplementary file 1 [file oncotarget-08-27093-s001.pdf]

Paeonol suppresses solar ultraviolet-induced skin inflammation by targeting T-LAK cell-originated protein kinase

SUPPLEMENTARY FIGURE

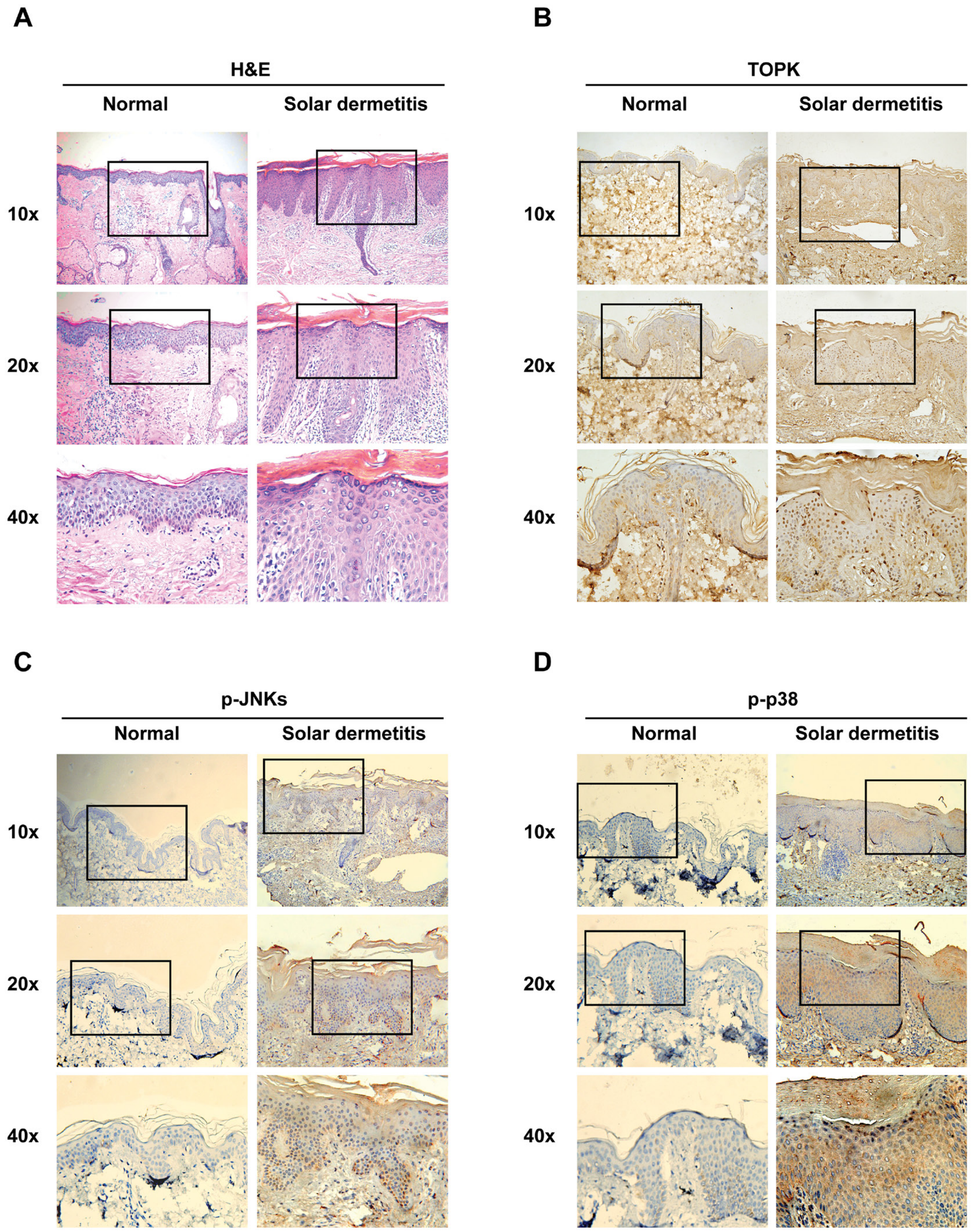

Supplementary Figure 1: The levels of TOPK, phosphorylation of p38 and JNKs are increased in human solar dermatitis. **A.** The pathological change of solar dermatitis compared with the normal skin was shown by H&E. **B, C** and **D.** Immunohistochemistry method was used to determine TOPK, phospho-p38 or phospho-JNKs in human solar dermatitis and normal skin tissues. The magnification of representative photos for H&E and the immunohistochemical staining are 10×, 20×, and 40× respectively.
